# Social concerns related to HIV status disclosure and participation in the prevention of mother-to-child transmission of HIV care among pregnant women in Kenya

**DOI:** 10.1186/s12884-020-02907-x

**Published:** 2020-04-16

**Authors:** Björn Nordberg, Erin E. Gabriel, Edwin Were, Eunice Kaguiri, Anna Mia Ekström, Anna Kågesten, Susanne Rautiainen

**Affiliations:** 1 Department of Global Public Health, Global and Sexual Health (GloSH), Karolinska Institutet, Stockholm, Sweden; 2 Department of Infectious Diseases, Helsingborg Hospital, Helsingborg, Sweden; 3 Department of Medical Epidemiology and Biostatistics, Karolinska Institutet, Stockholm, Sweden; 4 Department of Reproductive Health, Moi University, Eldoret, Kenya; 5 Partners in Prevention, Moi University, Eldoret, Kenya; 6 Department of Infectious Diseases, Karolinska University Hospital, Stockholm, Sweden; 7 Brigham and Women’s Hospital, Boston, USA

**Keywords:** HIV, Concerns, Disclosure, Pregnant women, PMTCT, Kenya

## Abstract

**Background:**

Social concerns about unintentional HIV status disclosure and HIV-related stigma are barriers to pregnant women’s access to prevention of mother-to-child transmission of HIV (PMTCT) care. There is limited quantitative evidence of women’s social and emotional barriers to PMTCT care and HIV disclosure. We aimed to investigate how social concerns related to participation in PMTCT care are associated with HIV status disclosure to partners and relatives among pregnant women living with HIV in western Kenya.

**Methods:**

A cross-sectional study, including 437 pregnant women living with HIV, was carried out at enrolment in a multicentre mobile phone intervention trial (WelTel PMTCT) in western Kenya. Women diagnosed with HIV on the day of enrolment were excluded. To investigate social concerns and their association with HIV disclosure we used multivariable-adjusted logistic regression, adjusted for sociodemographic and HIV-related characteristics, to estimate odds ratios (OR) and 95% confidence intervals (CI).

**Results:**

The majority (80%) had disclosed their HIV status to a current partner and 46% to a relative. Older women (35–44 years) had lower odds of disclosure to a partner (OR = 0.15; 95% CI: 0.05–0.44) compared to women 18–24 years. The most common social concern was involuntary HIV status disclosure (reported by 21%). Concern about isolation or lack of support from family or friends was reported by 9%, and was associated with lower odds of disclosure to partners (OR = 0.33; 95% CI: 0.12–0.85) and relatives (OR = 0.37; 95% CI: 0.16–0.85). Concern about separation (reported by 5%; OR = 0.17; 95% CI: 0.05–0.57), and concern about conflict with a partner (reported by 5%; OR = 0.18; 95% CI: 0.05–0.67), was associated with lower odds of disclosure to a partner.

**Conclusions:**

Compared to previous reports from Kenya, our estimated disclosure rate to a partner is higher, suggesting a possible improvement over time in disclosure. Younger pregnant women appear to be more likely to disclose, suggesting a possible decreased stigma and more openness about HIV among younger couples. Healthcare providers and future interventional studies seeking to increase partner disclosure should consider supporting women regarding their concerns about isolation, lack of support, separation, and conflict with a partner. PMTCT care should be organized to ensure women’s privacy and confidentiality.

## Background

Worldwide in 2018, about 19 million women aged 15 years or older were living with HIV [1] and an estimated five hundred children per day were newly infected with HIV, mainly due to mother-to-child transmission [1]. In Sub-Saharan countries including Kenya, Ethiopia, Uganda and the United Republic of Tanzania, an estimated 38% of new HIV infections among children were attributed to pregnant and breastfeeding women not being retained on antiretroviral therapy (ART) in 2018 [2]. In addition, research shows that by increasing the access and sustained adherence to ART, maternal health outcomes [3] and life expectancy of women living with HIV can be improved [3, 4].

In Kenya, an estimated 910,000 women aged 15 years or older were living with HIV as of 2018 [1], with data indicating that HIV was transmitted from mother-to-child in 12% of HIV exposed children in 2018 [2]. Since mother-to-child transmission is still unacceptably high, it is important to identify factors that can improve ART adherence among pregnant women and outcomes of prevention of mother-to-child transmission of HIV (PMTCT) care. One such factor is HIV status disclosure, which has been linked to increased ART adherence [5, 6] as well as facility delivery [5, 7], early infant HIV testing [8], infant antiretroviral prophylaxis [9, 10], and adherence to infant feeding guidelines [11].

PMTCT care as well as ART to prevent sexual transmission, condom use, and voluntary male medical circumcision are successful strategies for HIV prevention in low and middle-income countries [2, 3, 12]. However, qualitative evidence from sub-Saharan Africa (SSA) and Asia has shown that concerns about unintentional HIV status disclosure and HIV related stigma are barriers to participate in PMTCT care among pregnant women [13–18]. Qualitative studies have also identified other important barriers to HIV status disclosure including concerns about separation or divorce [19–23], conflicts with a partner [19, 21], intimate partner violence (IPV) [19–21, 23] and stigma in the family or community [20, 23]. However, only a few quantitative studies have been conducted to further identify social concerns related to HIV disclosure and participation in PMTCT care. A recent cross-sectional study conducted in Uganda found that concerns about abandonment, being beaten, and loss of financial support were the most prevalent reasons for non-disclosure to a partner [24]. Other studies from Zimbabwe, South Africa, and Barbados have estimated that women were less likely to disclose their HIV status if they were concerned about being divorced [25], subjected stigma [26] and abnormal reactions [27].

It is important to increase the understanding among healthcare workers and policymakers of pregnant women’s social and emotional barriers to HIV disclosure and participation in PMTCT care. This knowledge could be used to improve counselling in PMTCT care, improve women’s health and reduce mother-to-child transmission of HIV. The aim of this study is therefore to investigate how social concerns related to participation in PMTCT care are associated with HIV status disclosure to partners and relatives among pregnant women living with HIV in western Kenya.

## Methods

### Study design, population, and location

**Fig. 1:**
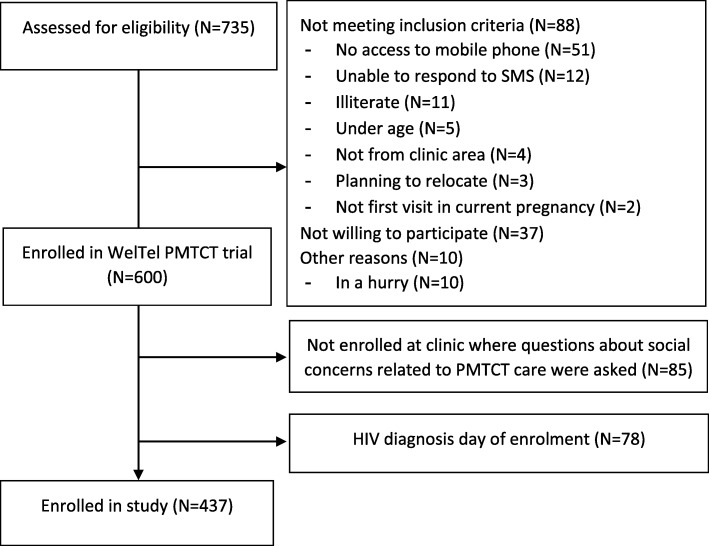
Flow chart of screening, enrolment, reasons for non-participation and selection of participants. PMTCT, Prevention of mother-to-child transmission of HIV

This is a cross-sectional study of pregnant women living with HIV and enrolled in the WelTel PMTCT trial from June 2015 to July 2016. The WelTel PMTCT trial is a multicentre randomised controlled trial designed to test the efficacy of weekly interactive mobile text messages for improving retention in PMTCT care among women and infants in western Kenya [28]. Pregnant women living with HIV, 18 years or older, consecutively presenting for their first antenatal care (ANC) visit in their current pregnancy were informed about the WelTel PMTCT trial by a trained local research assistant at their clinic. In order to be included, women had to own or have access to a mobile phone, be able to send text messages or have someone in close contact who could respond to text messages, and plan to be a resident of the clinic catchment area up to 24 months post-delivery. Established HIV infection was defined as two repeated Determine or Colloidal Gold tests for women newly diagnosed, or referral from HIV comprehensive care clinic for those with known HIV infection. Out of 735 women screened for trial participation, 600 women were enrolled in the WelTel PMTCT trial (Fig. 1) at six ANC clinics.

For our study, we used data from four out of the six ANC clinics in WelTel PMTCT, including a total of 515 women. At these four ANC clinics, located in Usain Gishu and Trans Nzoia counties, an enrolment survey collected information on social concerns related to participation in PMTCT care, in addition to other questions of HIV status disclosure, HIV testing, date and year of HIV diagnosis, barriers for accessing healthcare as well as mobile phone access and use that were asked at all six sites. Data were collected in interviews with trained fieldworkers using a structured questionnaire developed for this study (Additional file 1). Women diagnosed with HIV on the day of enrolment had no opportunity of having disclosed their HIV status and were therefore excluded from this analysis, leaving a study population of 437.

At the time of the enrolment, all clinics had adopted the World Health Organization’s (WHO’s) PMTCT Option B+ guidelines (i.e., life-long ART offered to all pregnant women living with HIV) [29]. All participants were informed that participation in the study was voluntary and that non-participation or withdrawal of consent would not affect the care. Women were interviewed face-to-face in a private space at the local ANC clinic to ensure participants’ comfort and confidentiality. All women provided informed consent, and the trial was approved by the Institutional Research and Ethics Committee at Moi University, Kenya (FAN: IREC 1292), and the Regional Ethics Committee, Stockholm, Sweden (2018/742–31/1).

### Measures

#### Sociodemographic characteristics

Women were interviewed about several lifestyle and social factors such as age at enrolment, educational status, marital status, cohabitation status, working status, and economic responsibility to other people. A current relationship was defined as being married or cohabitating with a husband/partner. Educational status was categorized as primary schooling or less, secondary schooling, or higher than secondary education. Working status was categorized as working outside the household (i.e., employed, self-employed, casual labour, farm work) or not working outside the household (i.e., unemployed, homemaker, student).

#### Disclosure of HIV status

The participants were asked if they had disclosed their HIV status and, if so, to whom: a partner (i.e., spouse or steady sexual partner); a relative (i.e., mother, father, sister, brother, child, or another relative); or another person.

#### Social concerns related to participation in PMTCT care

Questions about social concerns related to participation in PMTCT care were developed based on previous qualitative research of issues related to HIV status disclosure, uptake and retention in PMTCT care in Kenya [30–32]. The questionnaire was also developed in consultation with experienced local PMTCT providers in western Kenya. A pilot study was conducted prior to the study to test the feasibility of the questionnaire among pregnant women enrolled in PMTCT care in western Kenya. The participants were asked if they were ever concerned that attending HIV care or taking ART would lead to involuntary disclosure of HIV status (yes/uncertain/no). They were also asked if they were concerned that attending PMTCT visits or taking ART would lead to them being a burden or source of concern for others (yes/no); them losing the respect of family or community (yes/no); them being isolated or lacking support from family or friends (yes/no); them being teased or insulted (yes/no); them being separated from a partner (defined as separation, divorce or abandonment from spouse or partner, yes/no); them having a conflict with a partner (yes/no); them losing customers or losing a job (yes/no); them being a victim of IPV (defined as beating or other forms of physical violence by spouse or partner, yes/no); them having a child taken away (yes/no); or property taken away (yes/no).

### Statistical analysis

The associations between social concerns and HIV status disclosure to a partner or a relative were assessed by calculating odds ratios (OR) and 95% confidence intervals (CI) using multivariable-adjusted logistic regression. Based on previous literature of factors associated with HIV status disclosure, ORs were adjusted for age [33, 34], educational level [35], marital- and cohabitation status [26, 36–38], working status [39], time since HIV diagnosis [33], living with relatives [40] and economic situation [19, 26, 33]. A variable was excluded from the logistic regression model if a response alternative was reported by < 10 subjects (excluding concerns about IPV, a child taken away and property taken away). Fourteen women chose not to respond to the question about concern of involuntary HIV status disclosure, and those subjects were excluded when calculating the prevalence of that specific concern. They were also excluded from the logistic regression analysis. We further investigated whether ANC clinic of enrolment attenuated the observed findings by calculating both unadjusted and adjusted OR and 95% CI including ANC clinic of enrolment. All statistical analyses were performed using SPSS (IBM Corp. Released in 2016. IBM SPSS Statistics for Windows, Version 24.0. Armonk, NY: IBM Corp).

## Results

The characteristics of the 437 pregnant women are presented in Table 1. Women enrolled were 18–44 years old and the mean age was 29.9 years (SD = 5.7 years). As can be seen in Table 1, the majority reported that they were in a current relationship. Women who were not in a relationship were single (13.7%), divorced or separated (3.2%), or widowed (1.8%). The mean time since HIV diagnosis was 53.2 months (SD = 47.5).

**Table 1:** Characteristics of pregnant women admitted to PMTCT at first antenatal care visit (*N* = 437)

Characteristic	N (%)
**Age (years)**	
18–24	84 (19.2)
25–29	122 (27.9)
30–34	123 (28.1)
35–44	108 (24.7)
**Highest education**	
≤ Primary schooling	195 (44.6)
Secondary schooling	166 (38.0)
Higher education	76 (17.4)
**In a current relationship**^**a**^	355 (81.2)
**Living with relatives**	97 (22.2)
**Working outside the household**	179 (41.0)
**Economic responsibility**	
Only for herself	247 (56.5)
For more people than herself	190 (43.5)
**Time since HIV diagnosis**	
< 6 months	89 (20.4)
≥ 6 months	348 (79.6)

*PMTCT* Prevention of mother-to-child transmission of HIV

^a^ In a current relationship i.e. married or cohabitating with a husband/partner

### Disclosure of HIV status

**Fig. 2:**
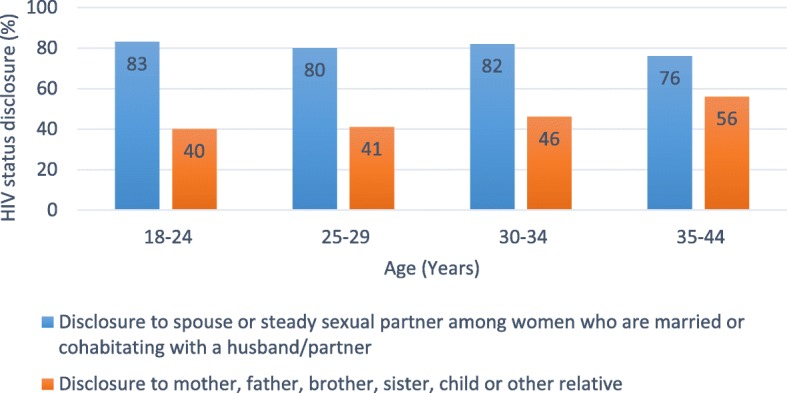
Prevalence of HIV status disclosure and age at enrolment in PMTCT care (*N* = 437), PMTCT, Prevention of mother-to-child transmission of HIV.

HIV status disclosure to a relative was reported by 202 (46.2%) and to another person by 34 (7.8%). Among the 355 women in a current relationship, 284 (80.0%) had disclosed to a partner. Figure 2 shows the prevalence of HIV status disclosure in relation to womens’ age.

### Social concerns among pregnant women at enrolment in PMTCT care

**Fig. 3:**
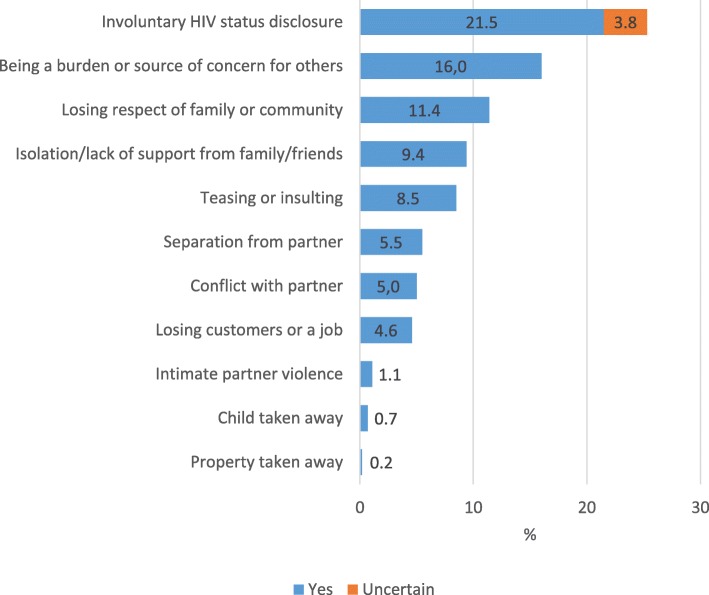
Prevalence of social concerns among pregnant women at enrolment in PMTCT care (*N* = 437). PMTCT, Prevention of mother-to-child transmission of HIV. Fourteen participants chose to not respond to the question about concern of involuntary HIV status disclosure, and they were excluded when the prevalence of that specific concern was calculated

Pregnant womens’ social concerns at enrolment in PMTCT care are presented in Fig. 3. As can be seen in Fig. 3, the most prevalent social concern, was that of involuntary HIV status disclosure. This was followed by the concern of being a burden or source of concern for others, losing respect of family or community, isolation or lack of support from family or friends, teasing or insulting, separation from partner, conflict with partner, losing customers or a job, intimate partner violence, having a child taken away, and property taken away.

### Social concerns and HIV status disclosure to partner

Table 2 shows the association of social concerns and HIV disclosure to a partner and a relative. As shown in Table 2, concerns that participation in PMTCT care would lead to separation from a partner, conflict with a partner, or isolation or lack of support from family or friends were all associated with significantly lower odds of having disclosed HIV status to a partner. Women aged 25–29 years (OR 0.30; 95% CI: 0.11–0.81), 30–34 years (OR 0.30; 95% CI: 0.11–0.86) and 35–44 years (OR 0.15; 95% CI: 0.05–0.44) had significantly lower odds of having disclosed to a partner as compared to women aged 18–24 years. In addition, women diagnosed with HIV < 6 months before enrolment compared with HIV diagnosis ≥6 months (OR 0.19; 95% CI: 0.09–0.39) had statistically significantly lower odds of having disclosed to a partner.

**Table 2:** Social concerns and association with HIV status disclosure (*N* = 423)

Disclosure of HIV status to:	%	Partner^a^ OR (95% CI)	Relative^b^ OR (95% CI)
Concern that attending PMTCT visits or taking			
ART would lead to:			
Involuntary HIV status disclosure^c^			
Yes	21.5	0.75 (0.39–1.44)	1.02 (0.60–1.71)
Uncertain	3.8	0.62 (0.14–2.70)	1.69 (0.53–5.41)
No	74.7	1.00 (ref.)	1.00 (ref.)
Being a burden or source of concern for others			
Yes	16.1	0.87 (0.38–1.98)	1.17 (0.63–2.16)
No	83.9	1.00 (ref.)	1.00 (ref.)
Losing respect of family or community			
Yes	11.3	0.95 (0.34–2.63)	0.62 (0.29–1.30)
No	88.7	1.00 (ref.)	1.00 (ref.)
Isolation or lack of support from family or friends			
Yes	9.5	0.33 (0.12–0.85)	0.37 (0.16–0.85)
No	90.5	1.00 (ref.)	1.00 (ref.)
Teasing or insulting			
Yes	8.0	2.09 (0.62–7.06)	0.93 (0.42–2.09)
No	92.0	1.00 (ref.)	1.00 (ref.)
Separation^d^ from partner			
Yes	5.4	0.17 (0.05–0.57)	0.44 (0.15–1.31)
No	94.6	1.00 (ref.)	1.00 (ref.)
Conflict with partner			
Yes	5.0	0.18 (0.05–0.67)	1.73 (0.58–5.15)
No	95.0	1.00 (ref.)	1.00 (ref.)
Losing customers or a job			
Yes	4.7	0.42 (0.11–1.66)	1.00 (0.36–2.76)
No	95.3	1.00 (ref.)	1.00 (ref.)

*PMTCT* Prevention of mother-to-child transmission of HIV, *ART* Antiretroviral therapy, *OR* Odds ratio, *CI* Confidence interval

^a^ Partner i.e. spouse or steady sexual partner

^b^ Relative i.e. mother, father, brother, sister, child or other relative

^c^ This question was asked more broadly to include general HIV care

^d^ Separation i.e. separation, divorce or abandonment

Multivariable logistic regression model adjusted for age, education, marital-and cohabitation status, employment status, economic responsibility to other people, living with relatives, and time since HIV diagnosis

### Social concerns and HIV status disclosure to relative

Concern that participation in PMTCT care would lead to isolation or lack of support from family or friends was associated with significantly lower odds of having disclosed to a relative (Table 2). The other social concerns related to PMTCT care investigated were not associated with disclosure to a relative. In addition, women in a current relationship (OR 0.35; 95% CI: 0.19–0.64) and women diagnosed with HIV < 6 months compared to ≥6 months before enrolment (OR 0.20; 95% CI: 0.10–0.39) had significantly lower odds of having disclosed HIV status to a relative. Further adjustments, including ANC clinic of enrolment, provided similar results.

## Discussion

In this cross-sectional study of pregnant women enrolled in PMTCT care in western Kenya, we observed that women who had not disclosed to a partner were more likely to have concerns about conflict, separation, and isolation or lack of support from family or friends. A high proportion had disclosed their HIV status to a current partner, and women < 25 years of age were more likely to have disclosed their HIV status to a partner compared to women ≥25 years. The most prevalent social concern was involuntary HIV status disclosure.

Our study is, to our knowledge, the first quantitative study to assess social concerns related to participation in PMTCT Option B+ care and their association with HIV disclosure among pregnant women living with HIV. We found that concern about isolation or lack of support from family or friends appear to be barriers to HIV disclosure, which is in line with a recently published mixed-methods study from Tanzania where stigma in family and community was the most frequently cited reason for non-disclosure of HIV status among 24 women in PMTCT care [23]. Our finding that concerns about conflict or separation appear to be a barrier for disclosure is also in line with one quantitative and five qualitative studies from SSA, suggesting that these concerns are important to address when developing strategies to promote HIV disclosure [19–23, 25].

The high proportion of partner disclosure is in line with recently published data from Kenya [39, 41], including western Kenya [42], suggesting that more women are disclosing their HIV status to their partners compared to earlier Kenyan studies [11, 36, 43–47]. In our study, younger women were more likely to have disclosed their HIV status to a partner compared to older women, indicating an increased openness and less stigma surrounding positive HIV status in younger couples in western Kenya, which could enable more male involvement in PMTCT care. While our results support those of a 2013 study of 250 pregnant women enrolled in PMTCT care in Tanzania [33], a 2017 cross-sectional study from South Africa found that younger women enrolled in PMTCT were less likely to disclose their HIV status to a partner [34]. In contrast to our study population, the majority of the women in the South African study were not married or cohabitating with a partner, and they used a different definition of being in a current relationship. A study published 2019 including 680 non-pregnant women living with HIV in Tanzania did not find age to be significantly associated with disclosure of HIV status [38]. The study included older women (the majority were 35–49 years old), and the age intervals tested in the analysis were different (the youngest age interval was 17–34 years).

Our findings suggest that future interventional studies to further increase partner disclosure among women in PMTCT care, such as counselling in support groups [48] or community interventions [49], should consider focusing on women ≥25 years and women diagnosed with HIV during the past 6 months. Therefore, continued counselling at routine follow-up visits could support and empower women in this effort, helping reduce concerns about isolation or lack of support from family and friends as well as concerns about conflict and separation from a partner. Counselling could, for example, involve peer-support [2, 49] and discussing what to do in worst-case scenarios.

We observed that involuntary HIV status disclosure related to participation in HIV care was the most common social concern among pregnant women in our study. In line with our results, previous qualitative studies from SSA and Asia have reported that concern about unintentional HIV status disclosure has been identified as a barrier to visiting PMTCT care facilities [13–18]. Concern about involuntary HIV disclosure has been suggested to limit access to care by promoting fear and isolation among pregnant women in Kenya [13]. Fear of breach of privacy and confidentiality have also been demonstrated to lead to loss to follow-up in PMTCT Option B+ care in Malawi [14]. In order to promote retention in care, it is therefore crucial that policymakers and health care workers organizes PMTCT care so that the risk of involuntary HIV disclosure is minimized when pregnant women visit their health care facilities. We observed that most of the concerns related to PMTCT care were reported by a low proportion of women which is in line with a UNAIDS report showing that the proportion of women and men reporting discriminatory attitudes toward people living with HIV in Kenya decreased from 28% in 2009 to 12% in 2014 [1]. The reduction in stigma is also supported by a 2017 mixed methods study from Uganda where a majority of women in PMTCT care felt that HIV-related stigma had reduced within their communities [20].

Key strengths of this study include a recent data collection, the large sample size, high participation rate, and multicentre design including both rural and urban centres from a large area in western Kenya. The study had several limitations. The study had an observational, cross-sectional design and we can, therefore, only assess associations but not causality. In addition, we cannot rule out reverse causality. WelTel enrolment was restricted to women with access to a mobile phone (93% of women screened for participation in the WelTel trial), possibly reducing the population of women to which we can generalize. However, in recent years the mobile penetration level has increased in Kenya [50]. It is also possible that women who declined to participate (5% of eligible women) did so because of social concerns or HIV related stigma, which would bias our prevalence estimates of social concerns related to participation in PMTCT care and HIV status disclosure. Finally, since our analysis was based on self-reports, risk of social desirability bias cannot be ruled out.

## Conclusions

Compared to previous reports from Kenya, our estimated disclosure rate to a partner is higher, suggesting a possible improvement over time in disclosure. Younger pregnant women appear to be more likely to disclose, suggesting a possible decreased stigma and more openness about HIV among younger couples. Healthcare providers and future interventional studies seeking to increase partner disclosure should consider supporting women regarding their concerns about isolation, lack of support, separation, and conflict with a partner. PMTCT care should be organized to ensure women’s privacy and confidentiality.

## Supplementary information

**Additional file 1.** Questionaire used in structured interviews.

## Data Availability

The datasets used and/or analysed during the current study are available from the corresponding author on reasonable request.

## Abbreviations

ANC: Antenatal care
ART: Antiretroviral therapy
IPV: Intimate partner violence
PMTCT: Prevention of mother-to-child transmission of HIV
SSA: Sub-Saharan Africa
WHO: World Health Organisation

## References

[1] UNAIDS. UNAIDS DATA 2019. https://www.unaids.org/en/resources/documents/2019/2019-UNAIDS-data. Accessed 9 Dec 2019.

[2] UNAIDS. Start Free Stay Free AIDS Free - 2019 report. https://www.unaids.org/sites/default/files/media_asset/20190722_UNAIDS_SFSFAF_2019_en.pdf. Accessed 9 Dec 2019.

[3] UNAIDS. On the Fast-Track to an AIDS-Free Generation. 2016. https://www.unaids.org/en/resources/documents/2016/GlobalPlan2016. Accessed 9 Dec 2019.

[4] Bor J, Herbst AJ, Newell ML, Barnighausen T. Increases in adult life expectancy in rural South Africa: valuing the scale-up of HIV treatment. Science (New York, NY). 2013;339(6122):961–5. 10.1126/science.1230413 PMC3860268 23430655

[5] Spangler SA, Onono M, Bukusi EA, Cohen CR, Turan JM. HIV-positive status disclosure and use of essential PMTCT and maternal health services in rural Kenya. J Acquir Immune Defic Syndr. 2014;67 Suppl 4:S235–42. 10.1097/QAI.0000000000000376 PMC4251910 25436823

[6] Watt MH, Cichowitz C, Kisigo G, Minja L, Knettel BA, Knippler ET (2019). Predictors of postpartum HIV care engagement for women enrolled in prevention of mother-to-child transmission (PMTCT) programs in Tanzania. AIDS Care.

[7] Sarko KA, Blevins M, Ahonkhai AA, Audet CM, Moon TD, Gebi UI (2017). HIV status disclosure, facility-based delivery and postpartum retention of mothers in a prevention clinical trial in rural Nigeria. Int Health.

[8] Hampanda KM, Nimz AM, Abuogi LL (2017). Barriers to uptake of early infant HIV testing in Zambia: the role of intimate partner violence and HIV status disclosure within couples. AIDS Res Ther.

[9] Sendo EG, Cherie A, Erku TA (2013). Disclosure experience to partner and its effect on intention to utilize prevention of mother to child transmission service among HIV positive pregnant women attending antenatal care in Addis Ababa. Ethiopia BMC Public Health.

[10] Kuonza LR, Tshuma CD, Shambira GN, Tshimanga M (2010). Non-adherence to the single dose nevirapine regimen for the prevention of mother-to-child transmission of HIV in Bindura town, Zimbabwe: a cross-sectional analytic study. BMC Public Health.

[11] Onono MA, Cohen CR, Jerop M, Bukusi EA, Turan JM (2014). HIV serostatus and disclosure: implications for infant feeding practice in rural South Nyanza, Kenya. BMC Public Health.

[12] GPC. Implementation of the HIV Prevention 2020 Road map - third Progress report, October 2019. https://www.unaids.org/sites/default/files/media_asset/22112019_UNAIDS_PCB45_CRP1_EN.pdf. Accessed 5 Feb 2020.

[13] Spangler SA, Abuogi LL, Akama E, Bukusi EA, Helova A, Musoke P (2018). From 'half-dead' to being 'free': resistance to HIV stigma, self-disclosure and support for PMTCT/HIV care among couples living with HIV in Kenya. Cult Health Sex.

[14] Cataldo F, Chiwaula L, Nkhata M, van Lettow M, Kasende F, Rosenberg NE, et al. Exploring the Experiences of Women and Health Care Workers in the Context of PMTCT Option B Plus in Malawi. J Acquir Immune Defic Syndr (1999). 2017;74(5):517–522. 10.1097/QAI.0000000000001273 PMC5340586 28045712

[15] Nzaumvila DK, Mabuza LH. Why do women not return for CD4 count results at Embhuleni Hospital, Mpumalanga, South Africa? Curationis. 2015;38(1). 10.4102/curationis.v38i1.1266. 10.4102/curationis.v38i1.1266 PMC6091793 26244457

[16] Schechter J, Bakor AB, Kone A, Robinson J, Lue K, Senturia K (2014). Exploring loss to follow-up among women living with HIV in prevention of mother to child transmission programmes in cote d'Ivoire. Global Public Health.

[17] Nguyen TA, Oosterhoff P, Ngoc YP, Wright P, Hardon A (2008). Barriers to access prevention of mother-to-child transmission for HIV positive women in a well-resourced setting in Vietnam. AIDS Res Ther.

[18] Kebaabetswe PM (2007). Barriers to participation in the prevention of mother-to-child HIV transmission program in Gaborone, Botswana a qualitative approach. AIDS Care.

[19] Duff P, Kipp W, Wild TC, Rubaale T, Okech-Ojony J (2010). Barriers to accessing highly active antiretroviral therapy by HIV-positive women attending an antenatal clinic in a regional hospital in western Uganda. J Int AIDS Soc.

[20] Naigino R, Makumbi F, Mukose A, Buregyeya E, Arinaitwe J, Musinguzi J (2017). HIV status disclosure and associated outcomes among pregnant women enrolled in antiretroviral therapy in Uganda: a mixed methods study. Reprod Health.

[21] Odiachi A, Erekaha S, Cornelius LJ, Isah C, Ramadhani HO, Rapoport L (2018). HIV status disclosure to male partners among rural Nigerian women along the prevention of mother-to-child transmission of HIV cascade: a mixed methods study. Reprod Health.

[22] Watt MH, Knippler ET, Knettel BA, Sikkema KJ, Ciya N, Myer L (2018). HIV disclosure among pregnant women initiating ART in Cape Town, South Africa: qualitative perspectives during the pregnancy and postpartum periods. AIDS Behav.

[23] Knettel BA, Minja L, Chumba LN, Oshosen M, Cichowitz C, Mmbaga BT (2019). Serostatus disclosure among a cohort of HIV-infected pregnant women enrolled in HIV care in Moshi, Tanzania: a mixed-methods study. SSM Popul Health.

[24] Ngonzi J, Mugyenyi G, Kivunike M, Mugisha J, Salongo W, Masembe S (2019). Frequency of HIV status disclosure, associated factors and outcomes among HIV positive pregnant women at Mbarara regional referral hospital, southwestern Uganda. Pan Afr Med J.

[25] Mucheto P, Chadambuka A, Shambira G, Tshimanga M, Gombe N, Nyamayaro W (2011). Determinants of nondisclosure of HIV status among women attending the prevention of mother to child transmission programme, Makonde district, Zimbabwe, 2009. Pan Afr Med J.

[26] Makin JD, Forsyth BW, Visser MJ, Sikkema KJ, Neufeld S, Jeffery B (2008). Factors affecting disclosure in south African HIV-positive pregnant women. AIDS Patient Care STDs.

[27] Kumar A, Waterman I, Kumari G, Carter AO (2006). Prevalence and correlates of HIV serostatus disclosure: a prospective study among HIV-infected postparturient women in Barbados. AIDS Patient Care STDs.

[28] Awiti PO, Grotta A, van der Kop M, Dusabe J, Thorson A, Mwangi J (2016). The effect of an interactive weekly mobile phone messaging on retention in prevention of mother to child transmission (PMTCT) of HIV program: study protocol for a randomized controlled trial (WELTEL PMTCT). BMC Med Inform Decis Mak.

[29] WHO. Programmatic Update: Use of Antiretroviral Drugs for Treating Pregnant Women and Preventing HIV Infection in Infants. 2012. https://www.who.int/hiv/pub/mtct/programmatic_update2012/en/. Accessed 9 Dec 2019.

[30] Awiti Ujiji O, Ekstrom AM, Ilako F, Indalo D, Rubenson B (2010). "I will not let my HIV status stand in the way." Decisions on motherhood among women on ART in a slum in Kenya- a qualitative study. BMC Womens Health.

[31] Awiti-Ujiji O, Mia Ekstrom A, Ilako F, Indalo D, Lukhwaro A, Wamalwa D (2011). 'Keeping healthy in the backseat': how motherhood interrupted HIV treatment in recently delivered women in Kenya. Afr J AIDS Res.

[32] Awiti Ujiji O, Ekstrom AM, Ilako F, Indalo D, Wamalwa D, Rubenson B (2011). Reasoning and deciding PMTCT-adherence during pregnancy among women living with HIV in Kenya. Cult Health Sex.

[33] Kiula ES, Damian DJ, Msuya SE (2013). Predictors of HIV serostatus disclosure to partners among HIV-positive pregnant women in Morogoro. Tanzania BMC Public Health.

[34] Adeniyi OV, Ajayi AI, Selanto-Chairman N, Goon DT, Boon G, Fuentes YO (2017). Demographic, clinical and behavioural determinants of HIV serostatus non-disclosure to sex partners among HIV-infected pregnant women in the eastern cape. South Africa PloS One.

[35] Erku TA, Megabiaw B, Wubshet M (2012). Predictors of HIV status disclosure to sexual partners among people living with HIV/AIDS in Ethiopia. Pan Afr Med J.

[36] Roxby AC, Matemo D, Drake AL, Kinuthia J, John-Stewart GC, Ongecha-Owuor F (2013). Pregnant women and disclosure to sexual partners after testing HIV-1-seropositive during antenatal care. AIDS Patient Care STDs.

[37] Olagbuji BN, Ezeanochie MC, Agholor KN, Olagbuji YW, Ande AB, Okonofua FE (2011). Spousal disclosure of HIV serostatus among women attending antenatal care in urban Nigeria. J Obstetr Gynaecol.

[38] Damian DJ, Ngahatilwa D, Fadhili H, Mkiza JG, Mahande MJ, Ngocho JS (2019). Factors associated with HIV status disclosure to partners and its outcomes among HIV-positive women attending care and treatment clinics at Kilimanjaro region. Tanzania PloS One.

[39] Kinuthia J, Singa B, McGrath CJ, Odeny B, Langat A, Katana A (2018). Prevalence and correlates of non-disclosure of maternal HIV status to male partners: a national survey in Kenya. BMC Public Health.

[40] Brou H, Djohan G, Becquet R, Allou G, Ekouevi DK, Viho I (2007). When do HIV-infected women disclose their HIV status to their male partner and why? A study in a PMTCT programme, Abidjan. PLoS Med.

[41] McGrath CJ, Singa B, Langat A, Kinuthia J, Ronen K, Omolo D (2018). Non-disclosure to male partners and incomplete PMTCT regimens associated with higher risk of mother-to-child HIV transmission: a national survey in Kenya. AIDS Care.

[42] Abuogi L, Hampanda K, Odwar T, Helova A, Odeny T, Onono M, et al. HIV status disclosure patterns and male partner reactions among pregnant women with HIV on lifelong ART in Western Kenya. AIDS Care. 2019:1–11. 10.1080/09540121.2019.1659915 PMC7056540 31488026

[43] Hardon A, Vernooij E, Bongololo-Mbera G, Cherutich P, Desclaux A, Kyaddondo D (2012). Women's views on consent, counseling and confidentiality in PMTCT: a mixed-methods study in four African countries. BMC Public Health.

[44] Tam M, Amzel A, Phelps BR (2015). Disclosure of HIV serostatus among pregnant and postpartum women in sub-Saharan Africa: a systematic review. AIDS Care.

[45] Irungu E, Chersich MF, Sanon C, Chege R, Gaillard P, Temmerman M (2012). Changes in sexual behaviour among HIV-infected women in west and east Africa in the first 24 months after delivery. AIDS (London, England).

[46] Moth IA, Ayayo AB, Kaseje DO. Assessment of utilisation of PMTCT services at Nyanza provincial hospital, Kenya. 2005;2(2):SAHARA J, 244–50. 10.1080/17290376.2005.9724847 17601006

[47] Bii SC, Otieno-Nyunya B, Siika A, Rotich JK (2008). Infant feeding practices among HIV infected women receiving prevention of mother-to-child transmission services at Kitale District hospital, Kenya. East Afr Med J.

[48] Mundell JP, Visser MJ, Makin JD, Kershaw TS, Forsyth BW, Jeffery B (2011). The impact of structured support groups for pregnant south African women recently diagnosed HIV positive. Women Health.

[49] Wanga I, Helova A, Abuogi LL, Bukusi EA, Nalwa W, Akama E, et al. Acceptability of community-based mentor mothers to support HIV-positive pregnant women on antiretroviral treatment in western Kenya: a qualitative study. BMC Pregnancy Childbirth. 2019;19(1):288. 10.1186/s12884-019-2419-z PMC6693232 31409297

[50] Kenya Cao. Fourth quarter sector statistics report for the financial year 2018/2019 (April-June 2019). https://ca.go.ke/wp-content/uploads/2019/09/Sector-Statistics-Report-Q4-2018-19.pdf. Accessed 9 Dec 2019.

