# Supplementary material for: Social concerns related to HIV status disclosure and participation in the prevention of mother-to-child transmission of HIV care among pregnant women in Kenya
Source: BMC Pregnancy Childbirth. 2020 Apr 16;20:225. doi: 10.1186/s12884-020-02907-x (PMC7164265; doi:10.1186/s12884-020-02907-x)
Supplement: Supplementary file 1 — Additional file 1. Questionaire used in structured interviews. [file 12884_2020_2907_MOESM1_ESM.pdf]

**Baseline Questionnaire**

**Date: ______________________OP / No /:______________Study ID :___________**

**I hereby confirm that the conditions of this study have been read to me and I agree to participate.**

**The answers you give on this form will be used to plan ways to help other people stay in care to prevent mother to child transmission of HIV. Please do your best to answer all the questions. If you do not wish to answer a question, please draw a line through it. If you do not know how to answer a question, ask your interviewer for help. Thank you for helping in this important study**.

Please check *one* of the options below.

***A. Socio-demographic characteristics***

**1. Date of birth _______________ or Age ______**

**2. What is the highest level of education you have completed?**

1. Never been to school ☐
2. Some primary school ☐
3. Primary school ☐
4. Some secondary school ☐
5. Secondary school ☐
6. Some tertiary-level schooling ☐
7. Tertiary/vocational school ☐
8. University ☐

**3. Which language can you read and write comfortably enough to text-message?**

1. Kiswahili ☐
2. English ☐
3. Both English and Kiswahili ☐
4. Another language ☐ Please specify:____________________
5. None ☐

**4. What is your marital status?**?

1. Married to one partner ☐
2. Married to more than one partner ☐
3. Widow. ☐
4. Single ☐
5. Divorced or separated ☐

**5a. How many people do you reside with, excluding yourself?**

1. 0 ☐ Go to item 6.
2. 1 ☐
3. 2-3 ☐
4. 4-5 ☐
5. ≥6 ☐

Of these, how many are children? ________

**5b.** What is the nature of relationship to those you reside with?
**(You may check more than one option)**

1. Husband /partner / ☐
2. Children ☐
3. Friends ☐
4. Relatives ☐
5. Other ☐ Please specify:____________________________

6. How many people are you supporting financially **(excluding yourself)?**

   1. 0 ☐
   2. 1 ☐
   3. 2-3 ☐
   4. 4-5 ☐
   5. 6-7 ☐
   6. ≥8 ☐

7. Are you living in the PMTCT clinic catchment area?

   1. Yes ☐
   2. No ☐ **If response is no, please specify where you live and skip the next question 7a and move to question 8.**

   ______________________________________________

   **7a.** How long have you been living in **(mentioned in number 7 before)?**
   Please specify: ____________days ________months **OR**_______ years

**8.** How long does it take you to reach the clinic from your residence?
Please specify: _________ minutes OR _______ hours

**9. How do you get to the clinic (main mode of transportation) e.g. walking, public transport etc.?**
Please specify ________________________________________

**10. How much do you usually pay for return travel to the clinic?**
Please specify _________________________________ KES

**11. What is your present occupation?**

1. Employed ☐
2. Self-employed ☐
3. Casual labour ☐
4. Unemployed ☐
5. Student ☐
6. A homemaker ☐
7. Other ☐ Please specify:___________________________

**If you are not currently working, please go to B**

**12.** How much time do you have to miss from work to attend the clinic?

1. Less than 2 hours ☐
2. Up to half a day ☐
3. A whole day ☐
4. More than one day a ☐

**13a. How much do you earn in a month?**

1. Less than 1000 ☐
2. KES 1000-5000 ☐
3. KES 5000-10,000 ☐
4. More than KES 10,000 ☐
5. Not willing to disclose ☐
6. Not certain ☐

**13b. What is your total <u>household income</u> per month?**

1. Less than KES 1000 ☐
2. KES 1000-5000 ☐
3. KES 5000-10,000 ☐
4. KES 10,000-20,000 ☐
5. More than KES 20,000 ☐
6. Not willing to disclose ☐
7. Not certain ☐

***B. Testing, disclosure, HIV care, social support***

**Where did you have your first positive HIV test done?**

1. At this facility ☐
2. Other **Please specify**: ____________________ ☐

**2.** When did you receive your first positive HIV test**?** ***dd______mm_______yyyy________***

**3.** For how long have you been attending CCC clinic?

1. This is my first visit to the clinic ☐
2. Less than 6 months ☐
3. 6 months to less than 1 year ☐
4. 1 to 2 years ☐
5. More than 3 years ☐

**4.** Overall, how do you feel about the HIV care that you have received so far at this clinic?
**Please circle one number.**

| Completely dissatisfied | Mostly dissatisfied | Somewhat dissatisfied | Mixed feelings / | Somewhat satisfied / | Mostly satisfied / | Completely satisfied / |
|---|---|---|---|---|---|---|
| 1 | 2 | 3 | 4 | 5 | 6 | 7 |

**5a.** If today is <u>not</u> the first day you found out you are HIV positive, have you disclosed your HIV status to anyone?

1. Yes ☐ (go to #5b)
2. No ☐ (**go to #6**)
3. Not applicable ☐ (**go to c**)

**5b.** If yes, who have you told about your status**?** ***(circle all that apply)***

| | | |
|---|---|---|
| Spouse or steady sexual partner | 1 | |
| Child | 2 | |
| Mother | 3 | |
| Other female relative | 4 | |
| Father | 5 | |
| Other male relative | 6 | |
| Sister | 7 | |
| Friend | 8 | |
| Brother | 9 | |
| Other | 10 | Please specify_________________________ |

**6.** How often is the following kind of support available to you if you need it? **Please circle one number.**

| Someone to turn to for suggestions about how to deal with a personal problem. | None of the time | A little of the time | Some of the time | Most of the time | All of the time |
|---|---|---|---|---|---|
| | 1 | 2 | 3 | 4 | 5 |

7. *We would now like to ask you a few questions about your experience with your HIV infection.*

| *To what extent do you disagree or agree with the following statements* | Strongly disagree | Disagree | Neutral | Agree | Strongly Agree |
|---|---|---|---|---|---|
| I am overwhelmed by the visit schedule | ☐ | ☐ | ☐ | ☐ | ☐ |
| I don't have any problems in adhering to my medication | ☐ | ☐ | ☐ | ☐ | ☐ |
| I am able to cope with my HIV infection | ☐ | ☐ | ☐ | ☐ | ☐ |

8. Were you ever worried that attending CCC or taking HIV medications may disclose your HIV status to people who you do not want to know about your HIV infection?

| | |
|---|---|
| Yes | (1) |
| No | (2) |
| Uncertain | (3) |

9. Has attending CCC or taking HIV medications actually caused a disclosure of your HIV status to people who you do not want to know about your HIV infections?

| | |
|---|---|
| Yes | (1) |
| No | (2 ) |
| Uncertain | (3) |

10. Are you worried that you may experience any of the following social problems from attending PMTCT visits or taking PMTCT medications? (***Circle all that apply***)

| | |
|---|---|
| Conflict with spouse or partner | 1 |
| Separation or divorce from spouse or partner | 2 |
| Abandonment by spouse or partner | 3 |
| Beating or other forms of physical violence by spouse or partner | 4 |
| Isolation and or lack of support from family or friends | 5 |
| Being a burden or source of worry for others | 6 |
| Teasing or insulting | 7 |
| Loss of respect or standing with the family and/or community | 8 |
| Loss of customers | 9 |
| Loss of a job | 10 |
| Taking away of property | 11 |
| Taking away of a child | 12 |

11. Have you actually experienced any of the social problems listed in question 10?

Yes ☐
No ☐

If yes, which problem did you experience**?** ____________________________________

______________________________________________________________________

*C. Cell phone access and use*

**1. Do you have a landline?**

1. Yes ☐ 2. No ☐

**Do you own a cell phone?**

1. Yes ☐
2. No ☐ If no, please go to **question 5.**

**1. If you own a phone, who shares access to your phone? (You may check more than one option)**

1. Husband / partner ☐
2. Another household member ☐ Please specify_______________
3. Non-household member ☐ Please specify _______________
4. Other ☐ Please specify _______________
5. No one ☐

**4. Do you switch your cell phone off during the day?**

1. Yes ☐ 2. No ☐ If no, please go to question 6**.**

**If yes, why do you turn your phone off?**

**_______________________________________________**

If yes, are there certain times your phone is usually turned on? **Please go to question 6.____________________________________**

**5.** <u>If you do not own a cell phone</u>, do you have regular access to somebody else's cell phone?

1. Yes ☐ 2. No ☐

**5a. If yes, whose phone do you have regular access to?**

1. Husband/partner ☐
2. Another household member ☐ Please specify _______________
3. Non-household member ☐ Please specify _______________
4. Phone kiosk ☐
5. Other ☐ Please specify_______________

**5b. Does the person you selected above (in question 5) know that you are HIV positive?**

1. Yes ☐ 2. No ☐

**6. Whose cell phone would you use to receive weekly text messages from the clinic? /**

1. My own phone ☐
2. My spouse or partner's phone ☐
3. Another household member's phone ☐ Please specify _______________
4. A non-household member's phone ☐ Please specify _______________
5. Other ☐ Please specify _______________
6. No one ☐

**7. Have you communicated with your healthcare provider by cell phone before?**

1. Yes ☐

2. No ☐

8. Would you prefer to communicate with your healthcare provider using a cell phone by….
**Please check one of the options below.**

1. Text message (SMS) ☐
2. Voice call ☐
3. Both would be fine ☐
4. I would prefer not to communicate with my healthcare provider using a cell phone. ☐

**9. Do you have any concerns about receiving text messages from your healthcare provider over your cell phone?**

1. Yes ☐
2. No ☐

**9b. If yes, please indicate your concerns.**

______________________________________________________________________________
______________________________________________________________________________
______________________________________________________________________________

**D**. ***Costing for accessing health services***

1. a) **In coming to ANC or PMTCT today, did you have to pay for: (tick all that apply)/**

**Transport (one way)** ☐
**Including child-care** ☐
**Food during visit** ☐
**Phone call/SMS** ☐
**Other** ☐ Please specify________________________________

1b) In coming to ANC or PMTCT today, how much did you pay for: **(tick all that apply)**

Transport (one way) ______KSh

Including child-care ______KSh
Food during visit ______KSh
Phone call/SMS ______KSh
Other ______KSh Please specify____________________________

2. Did you find it easy or difficult to incur these expenses? (***Circle one appropriate response***)

| | |
|---|---|
| Easy | (1) |
| Difficult | (2) |
| Neither easy nor difficult | (3) |
| Don't know | (4) |

3. What would you have been doing if you weren't at the clinic today? (***Circle all that apply***)

| | |
|---|---|
| Working for pay | (1) |
| Doing unpaid community work or volunteer work | (2) |
| Doing household chores such as cleaning, cooking, shopping for food, maintenance and repairs, working in the garden, gathering wood, gathering water, housework, etc | (3) |
| Taking care of children | (4) |
| Leisure activities (sport, watching TV, listening to music, reading, visiting friends and family, going to movies, etc.) | (5) |
| Attending school or other educational institution | (6) |
| Nothing *Skip to #6* | (7) |
| I don't know *Skip to #6* | (8) |
| Other, specify | (9) |

3 b) If you are working for pay, did you lose income from the time you took from your job to come here today?

Yes ☐
No ☐ ***skip option c***

3 c) **If yes,** how much money did you lose**?** _________________ KSh

4. Does attending ANC interfere with your ability to fulfill your role in your family?

Yes ☐
No ☐
Uncertain ☐

**Thank you so much for your participation!**

**Interviewer's initials** _________________
**Date of interview:** ____________________
